# Experimental Analyses of Spike Domains in Stress-Coated Grain-Oriented Electrical Steels (GOES)

**DOI:** 10.3390/s26092773

**Published:** 2026-04-29

**Authors:** Yusuke Kawamura, Helmut Pfützner, Georgi Shilyashki

**Affiliations:** 1 Magnet Test Labs, Vienna Magnetics GmbH, 1020 Vienna, Austria; 2 Institute of Biomedical Electronics, TU Wien, 1040 Vienna, Austria; 3 Steel Research Laboratories, Nippon Steel Corporation, Chiba 293-8511, Japan

**Keywords:** magnetic domain, spike domain, grain-oriented electrical steel, thermometry

## Abstract

Recent studies have revealed that spike domains can occupy a substantial fraction of the magnetic domain area in grain-oriented electrical steel (GOES), in some cases exceeding 30%. However, there is currently no established understanding of how spike domains influence the iron loss characteristics of GOES. To clarify this relationship, it is necessary to correlate local magnetic domain structures with corresponding local iron loss measurements. In this study, magnetic domain structures were observed using the Faraday effect, and local iron loss was measured with thermistors. By directly correlating these measurements, the influence of spike domains on iron loss was systematically investigated. The results indicate that the emergence of spike domains may contribute to a reduction in iron loss.

## 1. Introduction

Grain-oriented electrical steel (GOES) is a soft magnetic material used for transformer cores [1,2]. The magnetic properties of GOES are governed by its magnetic domain structure [3]. Observation of magnetic domains is crucial in the development of magnetic materials, and it is performed not only for microscale soft-magnetic systems such as nanowires [4,5,6,7,8] and thin films [9], but also for larger materials such as GOES.

One of the most important physical quantities in GOES is iron loss, which strongly affects the efficiency of electrical machines and power equipment, especially transformer cores [1]. Iron loss (also referred to as core loss or magnetic loss) refers to the energy dissipated as heat in a ferromagnetic material when subjected to an alternating magnetic field. The iron loss of GOES is strongly influenced by its magnetic domain structure [9].

The magnetic domain structure of GOES can be broadly classified into three types: main domains [9,10,11,12], lancet domains [13,14,15,16,17], and spike domains [18,19,20,21]. Among these, a main domain is a region bounded by 180° Bloch walls [9], and the spacing between these Bloch walls is known to be related to the anomalous eddy current loss in GOES [3,22]. A lancet domain is a structure that penetrates through the thickness of the GOES [13], and magnetostriction is generated during its formation and annihilation processes [23,24].

As both the main and lancet domains are closely related to key material properties—iron loss and magnetostriction—extensive studies have been conducted on these domain types.

In contrast, spike domains are known to form primarily near grain boundaries [9,16,25] and in regions where mechanical stress is applied, such as during domain refinement treatments [21,26,27,28]. Although several studies have reported the occurrence of spike domains and discussed their theoretical aspects [19], no prior work has clarified how spike domains influence the iron loss of GOES.

We have developed a technique that automatically classifies spike domains using a neural-network-based approach [29]. Analysis of GOES magnetic domain structures using this method revealed that, in some observed regions, spike domains accounted for more than 30% of the surface area of the GOES sheet. It should be noted that this result is based on observations from only one side of the sheet, and the actual proportion of spike domains may be higher when both sides are considered.

Although recent analyses have shown that spike domains can occupy a substantial portion of the magnetic domain structure of GOES, their influence on iron loss remains completely unknown. Clarifying the role of spike domains is therefore of great importance for the development of GOES. The aim of this study is to investigate how spike domains affect the iron loss of GOES by combining magnetic domain observation based on the Faraday effect with localized iron-loss measurements using thermistors.

## 2. Magnetic Domain Observation and Thermistor Measurement

**Figure 1:**
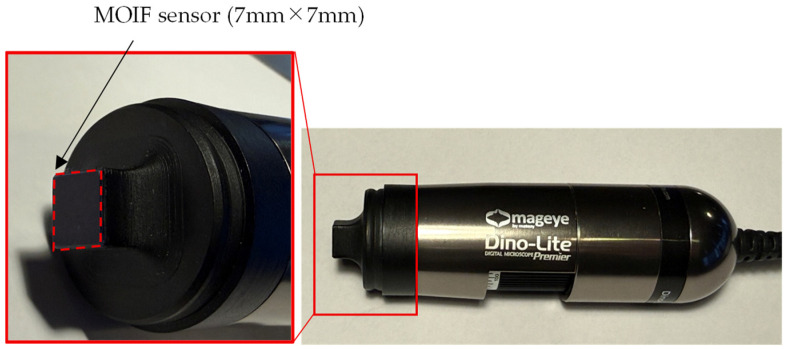
Photograph of the Mageye system.

To identify the regions where spike domains occur, we used the Mageye system (Matesy GmbH, Jena, Germany) for magnetic domain observation [30]. Figure 1 shows an overview of the Mageye system.

The device observes magnetic domain structures based on the Faraday effect. It provides magnetic domain images over an area of approximately 7 mm × 7 mm with a resolution of 2592 × 1944 pixels.

To enhance the clarity of the magnetic domain images obtained with the Mageye system, a calibration is performed. In this procedure, an image is first captured with the tip-mounted magneto-optical indicator film (MOIF) sensor not in contact with any surface. Subsequently, another image is acquired while the MOIF sensor is in contact with the GOES surface. The difference between these two images is then computed, and by adjusting the intensity threshold, a clearer magnetic domain image is obtained.

Next, the method for local iron-loss measurement is described. Spike domains can easily change their shape when tensile stress is applied to GOES [20]. Therefore, techniques that mechanically damage the tension coating, such as the needle method [31,32], are not suitable for local iron-loss evaluation. To avoid this issue, we adopt a heat-measurement-based approach in which the temperature rise generated during magnetic excitation of GOES is measured. Several techniques have been proposed to evaluate iron loss using the heat generated in GOES, including infrared-thermography-based measurements [33,34,35] and thermistor-based measurements [36,37,38,39,40,41]. Infrared thermography allows simultaneous measurement at multiple points; however, achieving high accuracy requires operating under vacuum conditions [33]. In contrast, thermistor-based measurements offer higher accuracy than infrared thermography, although the measurement is limited to a single point where the thermistor is in contact with the surface. According to [40], the estimation error can be kept within 2%.

In this study, we adopt the thermistor-based approach to measure local iron loss, as developed in 1982 by H. Pfützner [37]. According to [37], it shows the following characteristics:

- A large sample is magnetized in a thermal chamber, in comparison to a non-magnetized dummy sample.
- Miniature thermistors are mounted at the lower end of a glass tube for a minimum time constant.
- The sensor is loaded vertically with 10 g, for defined contact to the sample surface, during scanning of different crystallites of steel.
- Evaluation is restricted to a few seconds, for local testing.

**Figure 2:**
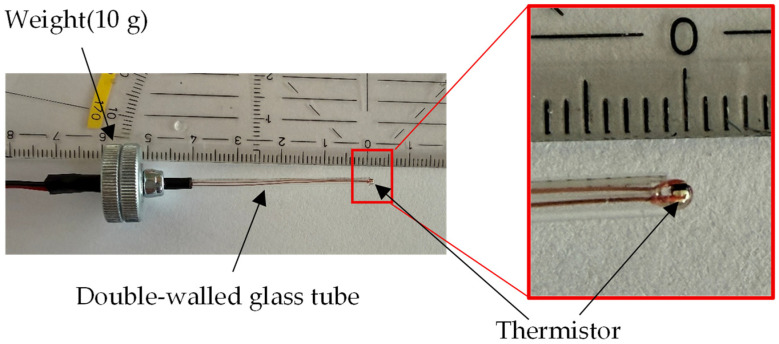
Thermistor probe.

Figure 2 shows the thermistor probe fabricated for this study. The thermistor probe was fabricated using the G10K3976 thermistor manufactured by TE Connectivity, Galway, Ireland. The probe consists of two thin glass tubes arranged concentrically to guide the two copper leads of the thermistor. To ensure stable contact with the GOES surface, a 10 g weight was incorporated into the probe structure.

**Figure 3:**
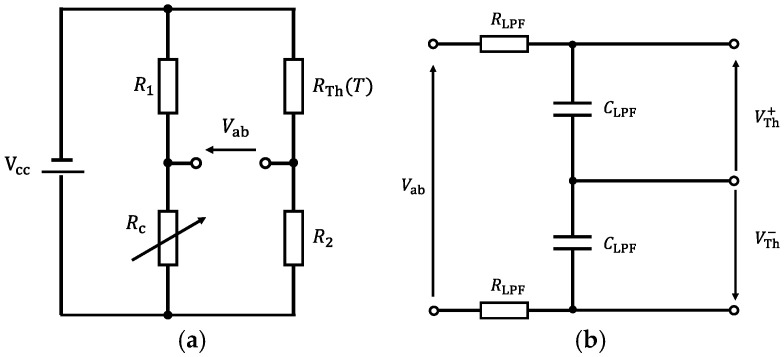
(**a**) Bridge circuit; (**b**) First-order LPF for anti-aliasing.

To convert the resistance change of the thermistor into an electrical signal, a thermistor bridge circuit was constructed. The thermistor bridge circuit is shown in Figure 3.

Figure 3a shows the thermistor bridge circuit. $R_{Th}(T)$ (Ω) represents the electrical resistance of the thermistor, which varies with temperature *T* (K). The fixed resistors $R_{1}$ and $R_{2}$ were set to 4.7 kΩ and 1.8 kΩ, respectively. $R_{c}$ is a variable resistor, and its resistance value was adjusted during measurement so that the bridge voltage $V_{ab}$ becomes 0 V.

Figure 3b shows the anti-aliasing filter applied to the thermistor bridge voltage $V_{ab}$. Since $V_{ab}$ was differentially acquired by the data acquisition device (USB-6210, Manufactured by National Instruments, Austin, TX, USA), identical RC filters were applied to $V_{Th}^{+}$ and $V_{Th}^{-}$, as illustrated in Figure 3b. The resistance and capacitance of the anti-aliasing filter, $R_{LPF}$ and $C_{LPF}$, were set to $R_{LPF}=10\;\mathrm{k\Omega}$ and $C_{LPF}=1\;\mu F$, respectively, resulting in a cutoff frequency of 15.9 Hz. The thermistor used in this study (G10K3976) has a thermal time constant of approximately 0.5 s, corresponding to a characteristic frequency of about 0.3 Hz. Since the cutoff frequency of the filter is sufficiently higher than this characteristic frequency, the filter does not significantly affect the measurement of the temperature variation.

**Figure 4:**
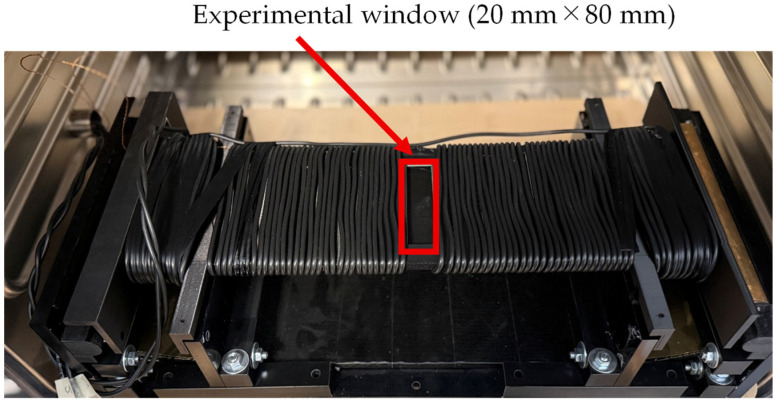
Single sheet tester (SST) with an experimental window.

For magnetic excitation of the GOES, a specifically adapted so-called multi frequency single sheet tester (MF-SST, Manufactured by Vienna Magnetics GmbH, Vienna, Austria) [42,43] with an experimental window [44], as illustrated in Figure 4, was used. As closer discussed in [44], the tester is characterized by the following:

- Compared to the standard SST with a sample size of 500 mm × 500 mm, a smaller size of 500 mm × 100 mm is used.
- Sample magnetization is not performed with a crystalline double yoke of SiFe, but by an amorphous single yoke from Fe-based foils, for dynamic induction patterns of frequencies up to 10 kHz.
- For defined control of spatial induction, magnetization is performed by two main coils and two booster coils close to the pole faces.
- Instead of applying the so-called current method, the field strength is detected directly by means of two printed tangential H-coils of about 150 mm × 100 mm each.
- Induction control is not performed by the conventional feed-back method for the whole period, but by digital synthetization of, e.g., n=5000 instantaneous values, e.g., allowing for a factor of 1.111.
- Loss evaluation is performed for n instantaneous values and for the corresponding—usually determined—mean value.
- The repeatability of the global iron loss measurement is within 0.3%, and the absolute accuracy is within 2% [43].

A key feature of the system is that an experimental window is located at the center of both the B-coil and the H-coil [44]. By placing the thermistor within this experimental window, the local iron loss generated during magnetic excitation of the GOES could be measured, while the overall iron loss of the entire sheet was simultaneously obtained.

Next, the signal-processing procedure for the measured $V_{Th}^{+}$ and $V_{Th}^{-}$ is described. In thermistor-based measurements, noise suppression is crucial because iron loss is estimated from extremely small temperature variations [40]. One possible approach to reducing noise is to magnetically excite the GOES for a sufficiently long duration so that a large temperature rise is obtained. However, under prolonged excitation, the spatial distribution of the generated heat becomes homogenized due to thermal diffusion, making it difficult to estimate the local iron loss. Therefore, when estimating local iron loss using a thermistor, measurements should preferably be performed within a short time interval [37].

Therefore, in this study, we apply a multirate sampling scheme together with a Kalman-filter-based noise-reduction method, enabling high-accuracy iron-loss estimation even under short-duration excitation.

First, the difference between the measured time-series signals $V_{Th}^{+}(t)$ and $V_{Th}^{-}(t)$ is defined as

(1) $$V_{Th}(t)=V_{Th}^{+}(t)-V_{Th}^{-}(t).$$

In this study, $V_{Th}(t)$ is sampled at the highest possible sampling frequency $f_{f}$. The acquired data are then averaged and downsampled to the lower sampling frequency $f_{s}$. This averaging operation performed prior to downsampling effectively behaves as a moving-average filter.

Next, a Kalman filter [45] is applied to the downsampled signal $V_{Th}(t)$. The state vector x(t) is defined as

(2) $$x\left(t\right)=\left[\begin{array}{c} {\hat{V}}_{Th}(t)\\ \frac{d{\hat{V}}_{Th}(t)}{dt} \end{array}\right].$$

Here, ${\hat{V}}_{Th}(t)$ represents the true value of the thermistor bridge voltage $V_{Th}(t)$. In this study, a constant-velocity model is adopted to describe the state transition between discrete sampling instants. This model is not intended to represent the full thermal dynamics of the system but rather to provide a local approximation of the temperature evolution. Because the sampling interval (10 ms) is sufficiently smaller than the thermal time constant of the thermistor (approximately 0.5 s), the exponential temperature response can be locally approximated as linear. Therefore, the constant-velocity model provides an adequate and robust approximation for estimating the temperature derivative. The system matrix A is given by

(3) $$A=\left[\begin{array}{cc} 1 & t_{s}\\ 0 & 1 \end{array}\right].$$

Here, $t_{s}$(s) denotes the sampling interval after downsampling, and it is given by $t_{s}=f_{s}^{-1}$. In this system, only the thermistor bridge voltage $V_{Th}(t)$ is observable; therefore, the observation matrix H is given by

(4) $$H=\left[\begin{array}{cc} 1 & 0 \end{array}\right].$$

Accordingly, the state-space model can be expressed as

(5) $$x\left(t\right)=Ax\left(t-t_{s}\right)+n_{p}\left(t\right),$$

(6) $$z(t)=Hx\left(t\right)+n_{o}(t).$$

Here, $n_{p}(t)$ and $n_{o}(t)$ denote the process noise and observation noise, respectively. In this study, the process noise is modeled as white acceleration. The covariance matrix Q of the process noise is given by

(7) $$Q=q_{var}\left[\begin{array}{cc} \frac{{t_{s}}^{4}}{4} & \frac{{t_{s}}^{3}}{2}\\ \frac{{t_{s}}^{3}}{2} & {t_{s}}^{2} \end{array}\right].$$

Thus, the covariance of the observation noise $n_{o}(t)$ is denoted by $r_{var}$. In the present Kalman filter design, only $q_{var}$ and $r_{var}$ serve as design parameters.

In this study, the Kalman filter is applied to the measured thermistor bridge voltage $V_{Th}(t)$ in an offline manner, allowing the use of information from all time indices t. Therefore, we employ the Rauch–Tung–Striebel (RTS) smoother [46], which incorporates not only past and present data but also future observations into the filtering process. By utilizing future information, the RTS smoother achieves stronger noise reduction than the conventional (forward-only) Kalman filter.

## 3. Estimation of Iron Loss from Thermistor Measurements

In this chapter, the method for calculating the local iron loss of GOES from the voltage variation measured using the thermistor bridge circuit shown in Figure 3 is described.

First, the thermistor resistance $R_{Th}(T)$ (Ω) in the bridge circuit of Figure 3a varies with temperature T (K). Accordingly, the bridge voltage $V_{ab}\;$(V) can also be expressed as a function of temperature T, and is given by

(8) $$V_{ab}\left(T\right)=V_{cc}\left(\frac{R_{c}}{R_{1}+R_{c}}-\frac{R_{2}}{R_{Th}\left(T\right)+R_{2}}\right).$$

If the variable resistor $R_{c}$ (Ω) is adjusted prior to measurement such that $V_{ab}=0$ V, then the measured bridge voltage $V_{ab}\;(T)$ can be interpreted as the voltage generated solely by the temperature change from the initial state.

Next, we consider the conversion from the measured voltage to the temperature variation. The conversion coefficient $k_{cc}\;$(V/K) from voltage change to temperature change can be defined by differentiating (8) with respect to temperature T, yielding

(9) $$k_{cc}=\frac{dV_{ab}\left(T\right)}{dT}.$$

The data acquisition card receives the filtered voltage $V_{Th}$ (V), to which the anti-aliasing filter has been applied. Since the cutoff frequency of this low-pass filter is sufficiently higher than the thermistor time constant, its influence can be regarded as negligible. The temperature rise $T_{rise}(t)$ in the GOES can then be computed from the measured voltage $V_{Th}(t)$ (V) using the conversion coefficient $k_{cc}$ as

(10) $$T_{rise}\left(t\right)=\frac{V_{Th}\left(t\right)}{k_{cc}}.$$

Next, we consider the thermal energy associated with the thermistor and the GOES. The thermistor is heated by the thermal energy generated through the iron loss of the GOES sample. By neglecting the contact thermal resistance between the thermistor and the GOES surface, as well as heat dissipation to the surrounding air, and by assuming that the thermistor and GOES share the same temperature, the total thermal energy $Q_{ext}(t)$ (J) generated by magnetic excitation is given by

(11) $$Q_{ext}\left(t\right)=\left(m_{GO}^{eff}c_{GO}+m_{Th}^{eff}c_{Th}\right)T_{rise}\left(t\right).$$

Here, $m_{GO}^{eff}$ (kg) and $m_{Th}^{eff}$ (kg) denote the effective masses of the heated regions of the GOES and the thermistor, respectively. Similarly, $c_{GO}$ (J/(kg·K)) and $c_{Th}$ (J/(kg·K)) represent the specific heat capacities of the GOES and the thermistor, respectively.

Next, the rate of change in thermal energy per unit time, $P_{ext}$ (W), is obtained by differentiating (11) with respect to time:

(12) $$P_{ext}=\frac{dQ_{ext}(t)}{dt}=\left(m_{GO}^{eff}c_{GO}+m_{Th}^{eff}c_{Th}\right)\frac{dT_{rise}(t)}{dt}.$$

By dividing (12) by the effective mass of GOES, $m_{GO}^{eff}$, the specific energy loss $P_{Local}^{*}$ (W/kg), which is commonly used for evaluating GOES, can be obtained as

(13) $$P_{Local}^{*}=\frac{P_{ext}}{m_{GO}^{eff}}=\left(c_{GO}+\frac{m_{Th}^{eff}}{m_{GO}^{eff}}c_{Th}\right)\frac{dT_{rise}(t)}{dt}.$$

However, as written, the specific loss $P_{Local}^{*}$ cannot be estimated unless the effective masses $m_{GO}^{eff}$ and $m_{Th}^{eff}$ are known. In this study, we assume that the effective heated volume of the GOES and that of the thermistor are identical due to thermal diffusion. Under this assumption, the ratio $m_{GO}^{eff}/m_{Th}^{eff}$ can be converted into the ratio of the densities of GOES and the thermistor, $\rho_{GO}$ (kg/m^3^) and $\rho_{Th}$ (kg/m^3^), respectively. Accordingly, (13) can be rewritten as

(14) $$P_{Local}^{*}=\left(c_{GO}+\frac{\rho_{Th}}{\rho_{GO}}c_{Th}\right)\frac{dT_{rise}(t)}{dt}.$$

The assumption of equal effective volumes of GOES and the thermistor is introduced as a simplified approximation to relate the effective mass ratio to material densities. This assumption does not imply that the actual heated volumes are physically identical, as the heat diffusion process depends on thermal properties and geometry. Rather, this approximation provides an effective parameterization for describing the local thermal response. Consequently, the iron loss estimated using (14) may significantly deviate from the actual iron loss. This discrepancy arises from several factors, including the neglect of the thermal contact resistance between the GOES and the thermistor, the omission of heat dissipation to the surrounding air, and the assumption that the effective heated volumes of the GOES sheet and the thermistor are identical. In practice, it is therefore desirable to calibrate the locally estimated iron loss obtained from (14). For calibration, a calibration coefficient $k_{cf}$ is derived using the ratio between the global iron loss $P_{Global}$ (W/kg) measured with the SST and the average of the locally estimated values $P_{Local}^{*}$ obtained at N different positions:

(15) $$k_{cf}=\frac{P_{Global}}{\frac{1}{N}\sum_{i=1}^{N}P_{Local,i}^{*}}$$

Using the calibration coefficient $k_{cf}$ derived above, the calibrated local iron-loss estimate $P_{Local}$ (W/kg) is given by

(16) $$P_{Local}=k_{cf}P_{Local}^{*}=k_{cf}\left(c_{GO}+\frac{\rho_{Th}}{\rho_{GO}}c_{Th}\right)\frac{dT_{rise}(t)}{dt}.$$

However, the thermistor contact region is influenced by heat conduction within the GOES and by the thermal contact condition between the thermistor and the specimen. Furthermore, the relationship between the true local loss and the measured signal is not necessarily strictly linear and may vary slightly depending on local thermal conditions. As a result, even when the calibration coefficient $k_{cf}$ is applied, these effects cannot be completely eliminated. In addition, due to heat conduction within the GOES, the measured temperature rise represents the average temperature within a finite effective volume rather than the temperature at a single point.

It should also be noted that the local iron loss $P_{Local}$ estimated by (16) represents the total iron loss generated in GOES, including both hysteresis loss and eddy current loss components [47].

## 4. Experimental Methods

In this chapter, the experimental procedure is described. In the previous chapter, the method for estimating the local iron loss $P_{Local}$ was presented. In that formulation, the time derivative of the temperature rise, $dT_{rise}(t)/dt$, plays a crucial role. However, the thermistor measurements contain various types of noise, and even with anti-aliasing filtering and Kalman filtering, it is difficult to sufficiently suppress the noise for practical use. Therefore, we propose a more practical method for obtaining $dT_{rise}(t)/dt$.

In this study, a period with no magnetic excitation is first introduced at the beginning of the measurement. By observing the temperature behavior during this interval, the heat dissipation caused by the temperature difference between the GOES and the surrounding environment, as well as the magnitude of external disturbances, can be estimated. After a prescribed duration, magnetic excitation is applied to the GOES. Since the flux density waveform is distorted during the initial excitation phase due to waveform adjustments, the data in this interval are not used. Once the excitation waveform reaches a steady state, the temperature rise $T_{rise}(t)$ of the GOES is monitored for a fixed period. The average temperature change rate during this interval is then computed, and the previously estimated disturbance is subtracted, yielding an estimate of $dT_{rise}(t)/dt$.

**Figure 5:**
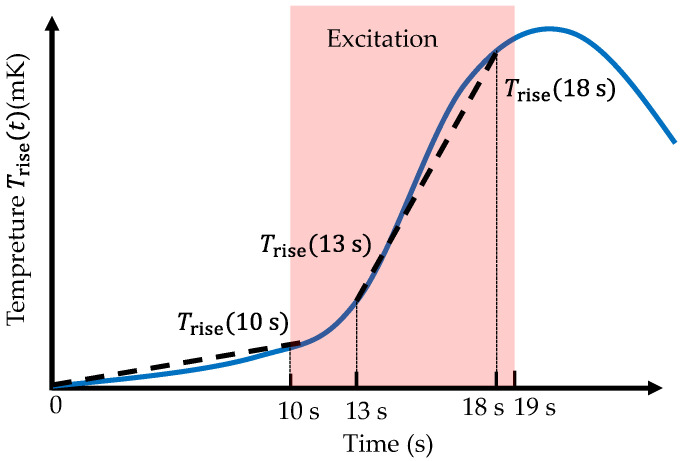
Illustration of the method for deriving the temperature rise.

The detailed procedure is illustrated in Figure 5. From the start of the experiment until 10 s, no magnetic excitation is applied. The average temperature slope during this initial interval (0–10 s) is computed. Subsequently, magnetic excitation is applied to the GOES at the prescribed frequency and flux density between 10 s and 19 s. Once the excitation waveform is considered stable—typically between 13 s and 18 s—the temperature slope over this 5 s interval is calculated. By subtracting the slope obtained during the 0–10 s interval from the slope during the 13–18 s interval, the temperature rise rate $dT_{rise}/dt$ can be determined as

(17) $$\frac{dT_{rise}(t)}{dt}\approx \frac{T_{rise}\left(18\;s\right)-T_{rise}\left(13\;s\right)}{5\;s}-\frac{T_{rise}\left(10\;s\right)-T_{rise}\left(0\;s\right)}{10\;s}.$$

The temperature rise during excitation is not strictly linear but reflects the transient thermal response of the system. In this study, the temperature rise rate is evaluated as the average slope over a finite time interval after the excitation waveform becomes stable. This approach provides a practical estimate of the effective heating rate while reducing sensitivity to measurement noise and transient fluctuations.

The GOES is magnetically excited at 100 Hz with a peak flux density of 1.7 T. In this study, excitation frequencies commonly used for standard GOES iron-loss evaluation, such as 50 Hz or 60 Hz, are not used. This is because lower excitation frequencies lead to smaller iron losses, resulting in smaller local heat generation and consequently smaller measured voltages from the thermistor, making the measurements more susceptible to noise. Therefore, a higher excitation frequency is employed to achieve a more accurate estimation. However, if the excitation frequency is increased excessively, the motion of the magnetic domain structure may differ from that observed at 50 Hz or 60 Hz [48,49,50]. Considering this trade-off, an excitation frequency of 100 Hz is selected as an appropriate balance. The purpose of this study is to experimentally evaluate the influence of spike domains on local iron loss, rather than to provide a direct quantitative assessment at commercial power frequencies such as 50 and 60 Hz. Further investigation across multiple excitation frequencies remains an important topic for future work.

In this study, the multirate sampling frequencies $f_{f}$ and $f_{s}$ were set to $f_{f}=20$ kHz and $f_{s}=100$ Hz, respectively. The design parameters of the Kalman filter, $q_{var}$ and $r_{var}$, were set to $q_{var}=10$ and $r_{var}=1$, respectively. Furthermore, thermistor measurements are highly sensitive to disturbances such as variations in ambient temperature and airflow. Therefore, the experiment was conducted inside a constant-temperature chamber in order to eliminate these external disturbances.

Prior to the measurements, the calibration coefficient $k_{cf}$ in (15) was determined. The GOES used in this study had a thickness of 0.23 mm. When this sheet was magnetically excited at 100 Hz and 1.7 T, the global iron loss was measured to be $P_{17/100}=2.23$ W/kg. Subsequently, within the experimental window shown in Figure 5, measurements were performed at 15 locations with a spacing of 5 mm, and each location was measured three times. Using (14), the locally estimated iron-loss values $P_{Local}^{*}$ were computed, and the average value was found to be 0.964 W/kg. Therefore, from (15), the calibration coefficient was obtained as $k_{cf}=2.31$. The constants used in the calculation of (14) were $c_{GO}=450$ J/(kg·K), $c_{Th}=800$ J/(kg·K), $\rho_{GO}=7650$ kg/m^3^, and $\rho_{Th}=2590$ kg/m^3^.

To investigate the influence of spike domains on the iron loss of GOES, it was necessary to carefully select the regions in which local iron loss was evaluated using the thermistor. To minimize differences in hysteresis loss and anomalous eddy-current loss, the evaluation regions were selected so that they had identical crystal orientation and similar main domain widths. Within such regions, areas in which spike domains appeared continuously were identified and compared, allowing the influence of spike domains to be estimated with minimal interference from other factors. In addition, to reduce residual shear strain introduced during sample cutting, the samples were annealed at 800 °C for 2 h in a nitrogen atmosphere prior to measurement.

**Figure 6:**
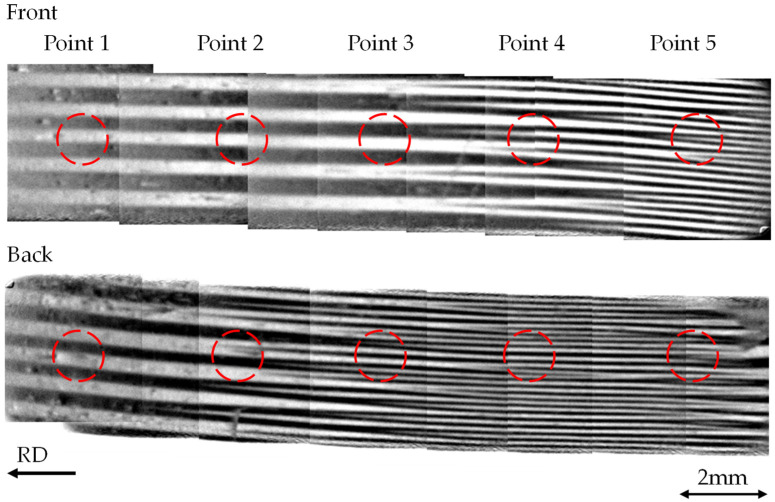
Magnetic domains on both surfaces of the region used for the local iron-loss measurement.

Figure 6 shows the magnetic domain images of the regions selected according to the conditions described above, confirming that the evaluated areas are located within the same grain and exhibit similar main domain widths. Figure 6 presents the domain structures on both sides of the GOES. These domain images were obtained using the Mageye system. Since the field of view of the Mageye is approximately 7 mm × 7 mm, multiple images were acquired and stitched together to cover the entire region. Within this stitched domain image, five equally spaced measurement points were selected, and the local iron loss at each point was evaluated using the thermistor. For the thermistor measurements, each point from Point 1 to Point 5 was measured five times, and the average value was used as the local iron loss for each point.

## 5. Experimental Results and Discussion

Focusing on Points 1–5 in Figure 6, no spike domain is observed on either surface at Point 1. At Point 2, the tip of a spike domain is present on the back surface, whereas no spike domain is observed on the front surface. At Point 3, spike domains occur frequently on the front surface, while the tip of a spike domain is observed on the back surface. At Points 4 and 5, spike domains occur frequently on both surfaces. In other words, the density of spike domains increases from Point 1 to Point 5. By correlating the measured local iron loss with the observed occurrence of spike domains, the influence of spike domains on the iron loss of the GOES can be estimated.

**Figure 7:**
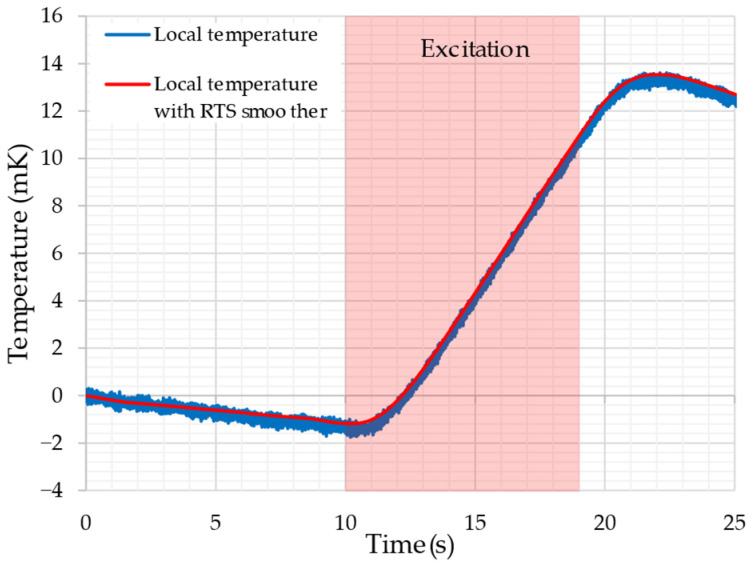
An example of experimental results $T_{rise}(t)$ (Point 1).

Figure 7 shows the measured temperature rise $T_{rise}(t)$ obtained using the thermistor. In this study, measurements were performed five times at each of the five measurement points; however, Figure 7 presents the result of the first measurement at Point 1. The results obtained in the other measurements exhibited waveforms similar to that shown in Figure 7. As explained in Chapter 4, no magnetic excitation was applied during the first 10 s after the start of the measurement. The GOES was then magnetically excited at 1.7 T and 100 Hz between 10 s and 19 s. It can be confirmed that the temperature of the GOES increases rapidly due to the magnetic excitation.

From the measurement results shown in Figure 7, the temperature rise rate $dT_{rise}(t)/dt$ was calculated using (17), and the local iron loss was then evaluated using (16). Table 1 lists the estimated iron-loss values obtained from five measurements at each point from Point 1 to Point 5.

In Table 1, “Maximum variation” represents the largest deviation calculated from the difference between the maximum and minimum values among the five measurements. Focusing on the “Maximum variation” for the five measurement points, the maximum fluctuation is approximately 1%, indicating that the local iron loss can be estimated with high reproducibility.

**Figure 8:**
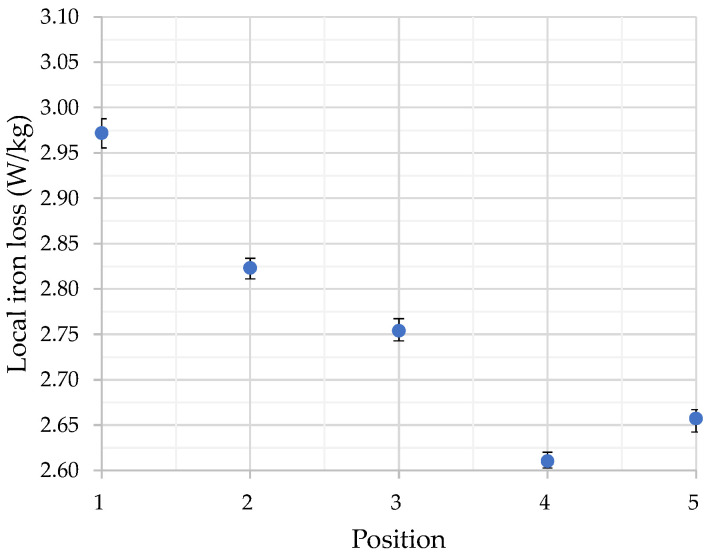
Average local iron loss at the five points.

Next, the averages at each point listed in Table 1 are plotted in Figure 8. The error bars in Figure 8 represent the “Maximum variation” values shown in Table 1.

As shown in Figure 8, Point 1 exhibits the highest iron loss, and the iron loss decreases significantly from Point 1 to Point 5. Although a slight increase is observed at Point 5 compared with Point 4, the overall trend shows lower iron loss in regions with higher spike domain density, considering that the variation at each point is limited to approximately 1% (Maximum variation). Comparing the magnetic domain in Figure 6 with the distribution of the local iron loss in Figure 8 reveals that Point 1, where no spike domain is observed, shows the highest iron loss. At Point 2, where the tip of a spike domain appears on the back surface, a reduction in iron loss is observed compared with Point 1. Similarly, as the density of spike domains increases, a further reduction in iron loss can be confirmed. From these results, it can be inferred that spike domains may contribute to the reduction of iron loss in the GOES. It should be noted that the thermistor measurements evaluate the total iron loss generated in GOES, including both hysteresis loss and eddy current loss. Therefore, the present method cannot distinguish whether the spike domain contributes primarily to the reduction in hysteresis loss or eddy current loss. Separation of these components may be possible by performing measurements at multiple frequencies and applying a loss separation method [47]. Such multi frequency measurements would not only enable separation of the loss components but also help assess the generality of the present findings beyond the 100 Hz condition, thereby providing stronger evidence that spike domains contribute to iron-loss reduction.

On the other hand, the thermistor measurements performed in this study are influenced by heat conduction from neighboring grains. Therefore, the measurements effectively represent the average iron loss over a relatively large area (e.g., approximately 10 mm×10 mm). Accordingly, the present thermistor measurements do not directly resolve individual micro-scale spike domains and instead represent the averaged local iron loss over a finite thermally affected region. The influence of thermal conduction can be reduced by shortening the measurement duration; however, doing so results in a smaller temperature change and consequently increases the variability of the measurement results. Therefore, in local iron-loss measurements using a thermistor, there exists a trade-off between the spatial resolution of the iron-loss measurement and the reproducibility of the measurement.

In previous studies using thermistor-based measurements [40], the excitation duration was 60 s, and the measurement variation was within 2%. In contrast, in this study, the measurement repeatability was improved to within 1% despite the much shorter excitation duration of 9 s. Therefore, compared with conventional thermistor-based measurements, the proposed method enables measurements with higher spatial resolution and improved reproducibility. To the best of the authors’ knowledge, for local measurements of GOES using a thermistor, the proposed method achieves the highest spatial resolution and reproducibility reported so far. This improvement is attributed to the multirate sampling and RTS smoothing based on the Kalman filter, the anti-aliasing filter applied to the bridge circuit, the suppression of external disturbances using a temperature-controlled chamber, and the proposed method for estimating the temperature rise rate.

In this study, it was concluded that spike domains may contribute to the reduction of iron loss in the GOES. However, to obtain more conclusive evidence, measurement methods other than the thermistor-based approach—such as the needle method [31,32] or measurements based on the Hall effect [51,52]—are considered to be more suitable. In addition, microstructure observations, such as optical microscopy, may provide additional insight into the relationship between spike domain formation and local iron loss.

## 6. Conclusions

This paper investigates the influence of spike domains on the iron loss of the GOES by conducting magnetic domain observations and local iron-loss measurements. For the local iron-loss measurement, a thermistor-based method was adopted. To improve the measurement accuracy, a noise-reduction approach combining an anti-aliasing filter, multirate sampling, and RTS smoothing based on a Kalman filter was proposed. By utilizing these filtering techniques, the measurement variation was limited to approximately 1% at most despite the short excitation duration, achieving significantly higher reproducibility than conventional thermistor-based measurement methods.

To correlate spike domains with the local iron-loss measurements, magnetic domain observations were conducted using an MOIF sensor. A region where the density of spike domains increases continuously was identified, and local iron-loss measurements were performed at equally spaced positions within this region. The results show that a significant reduction in iron loss occurs as the density of spike domains increases, suggesting that spike domains may contribute to the reduction of iron loss in the GOES.

The present measurements represent a first step toward evaluating the influence of spike domains, as the experiments were conducted using a single sample and a single excitation frequency. Future work should include measurements on multiple samples and at multiple excitation frequencies, enabling separation of hysteresis and eddy-current losses and a more general evaluation of the spike domain effect. In addition, the use of other measurement techniques, such as optical microscopy, the needle method, and Hall-effect-based measurements, would enable a more detailed investigation of spike domain effects.

## Figures and Tables

**Table 1:** Summary of measurement results at the five points (1.7 T/100 Hz).

	Point 1 (W/kg)	Point 2 (W/kg)	Point 3 (W/kg)	Point 4 (W/kg)	Point 5 (W/kg)
1	2.960	2.834	2.743	2.611	2.667
2	2.988	2.824	2.767	2.620	2.650
3	2.955	2.820	2.757	2.615	2.661
4	2.986	2.829	2.761	2.604	2.667
5	2.972	2.811	2.743	2.603	2.642
Average	2.972	2.824	2.754	2.611	2.658
Maximum variation	1.10%	0.80%	0.90%	0.68%	0.93%

## Data Availability

The data presented in this study are available from the corresponding author upon reasonable request. The data are not publicly available due to proprietary and confidentiality restrictions associated with the materials and experimental setup.

## Abbreviations

The following abbreviations are used in this manuscript:

GOES		Grain-oriented electrical steel
MOIF		Magneto-optical indicator film
RTS smoothing		Rauch–Tung–Striebel smoothing
T	K	Temperature
$R_{1}$, $R_{2}$	Ω	Resistance
$R_{c}$	Ω	Variable resistance
$R_{Th}(T)$	Ω	Thermistor resistance
$V_{cc}$, $V_{ab}$	V	Voltage
$R_{LPF}$	Ω	Resistance of the anti-aliasing filter
$C_{LPF}$	μF	Capacitance of the anti-aliasing filter
t	s	Time
$V_{Th}$, $V_{Th}^{+}$, $V_{Th}^{-}$	V	Thermistor-bridge voltage
$f_{f}$, $f_{s}$	Hz	Sampling frequency of the data acquisition card
$t_{s}$	s	Sampling time
x(t)	V, V/s	State vector (2 × 1 matrix)
${\hat{V}}_{Th}(t)$	V	True value of the thermistor bridge voltage
A		System matrix (2 × 2 matrix)
H		Observation matrix (1 × 2 matrix)
z(t)	V	Observed value
$n_{p}(t)$	V, V/s	Process noise matrix (2 × 1 matrix)
$n_{o}(t)$	V	Observation noise
Q		Covariance matrix of the process noise $n_{p}(t)$ (2 × 2 matrix)
$q_{var}$, $r_{var}$		Variance of the process noise and observation noise
$k_{cc}$	V/K	Conversion coefficient from the thermistor bridge voltage to temperature
$T_{rise}\left(t\right)$	K	Temperature rise in the GOES measured by the thermistor
$Q_{ext}\left(t\right)$	J	Thermal energy generated by the iron loss of the GOES
$m_{GO}^{eff}$, $m_{Th}^{eff}$	kg	Effective masses of the GOES sheet and the thermistor
$c_{GO}$, $c_{Th}$	J/(kg·K)	Specific heat of the GOES and the thermistor
$P_{ext}$	W	Thermal power generated by the magnetic excitation of the GOES
$P_{Local}^{*}$	W/kg	Uncalibrated local thermal power
$\rho_{GO}$, $\rho_{Th}$	kg/m^3^	Ratio of the densities of the GOES and thermistor
$P_{Global}$	W/kg	Global iron loss of the GOES
$k_{cf}$		Calibration coefficient
$P_{Local}$	W/kg	Calibrated local thermal power
